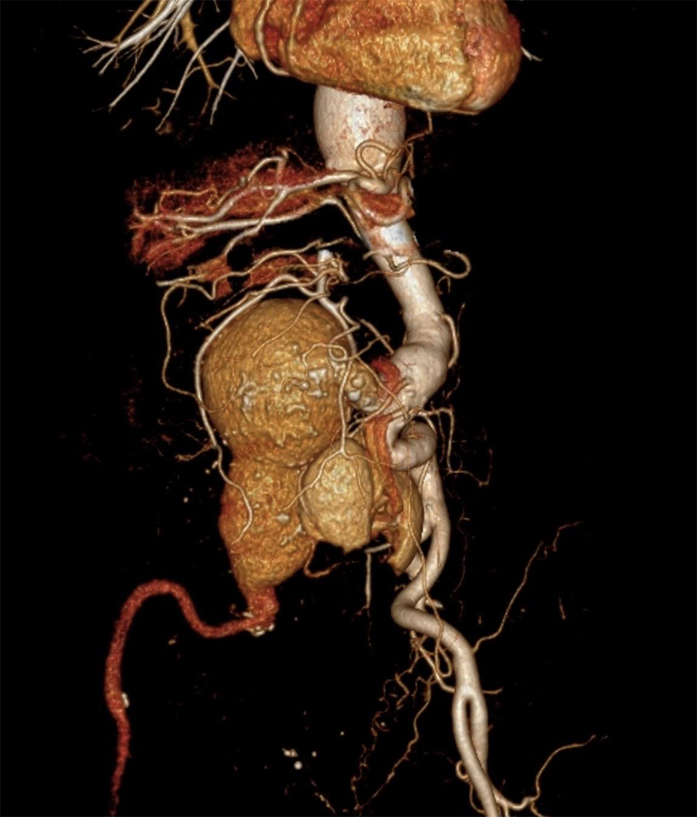# Symptomatic isolated right common iliac artery aneurysm in the setting of renal ectopia

**DOI:** 10.1016/j.jvscit.2022.06.021

**Published:** 2022-08-19

**Authors:** Thoetphum Benyakorn, Valeerat Swatesutipun, Benjamin W. Starnes

**Affiliations:** a Division of Vascular Surgery, Department of Surgery, Faculty of Medicine, Thammasat University, Pathum Thani, Thailand; b Division of Urology, Department of Surgery, Faculty of Medicine, Thammasat University Hospital, Thammasat University, Pathum Thani, Thailand; c Division of Vascular Surgery, Department of Surgery, School of Medicine, University of Washington, Seattle, WA

## Case report

An 80-year-old man presented with right lower abdominal pain for 1 day. Physical examination revealed tenderness and a pulsatile abdominal mass in the right lower quadrant. Computed tomography angiography showed an isolated right common iliac artery aneurysm with a maximum diameter of 69.2 mm and a right pelvic kidney (*A*/Cover). Two right renal arteries arising from the aorta supplied the pelvic kidney (*B*). A retrograde pyelogram is displayed in (*C)*. The anatomy was not suitable for endovascular devices. Therefore, we performed an open surgical repair of the right common iliac artery with a modified bifurcated graft due to size disproportion between proximal and distal anastomosis (*D*). The operation was successful, and the patient made a complete recovery. The patient approved the publication and all images.

## Discussion

A pelvic kidney is the most common type of renal ectopia, typically residing below the aortic bifurcation in the pelvis because of the failure of renal ascent during embryonic development.^1^ The incidence is 1 in 500 to 1200 live births.^2^ The most common renal ectopia is crossed fused renal ectopia with the orthotopic lower pole, but crossed unfused ectopia is very rare. Blood supply to the pelvic kidney can be derived from the distal aorta, aortic bifurcation, and/or iliac arteries. Renal artery anatomy can present challenges for aneurysm treatment. Open surgical repair may require renal artery re-implantation.^3^ In select anatomy, an endovascular iliac branch device can be used.^4^ The treatment of this condition should be tailored for each individual to attempt preservation of renal function.

The concomitant finding of an isolated common iliac artery aneurysm with crossed unfused renal ectopia with the renal pelvis is an exceedingly rare clinical entity. To the best of our knowledge, this is the first case report for this finding.

**Figure:**
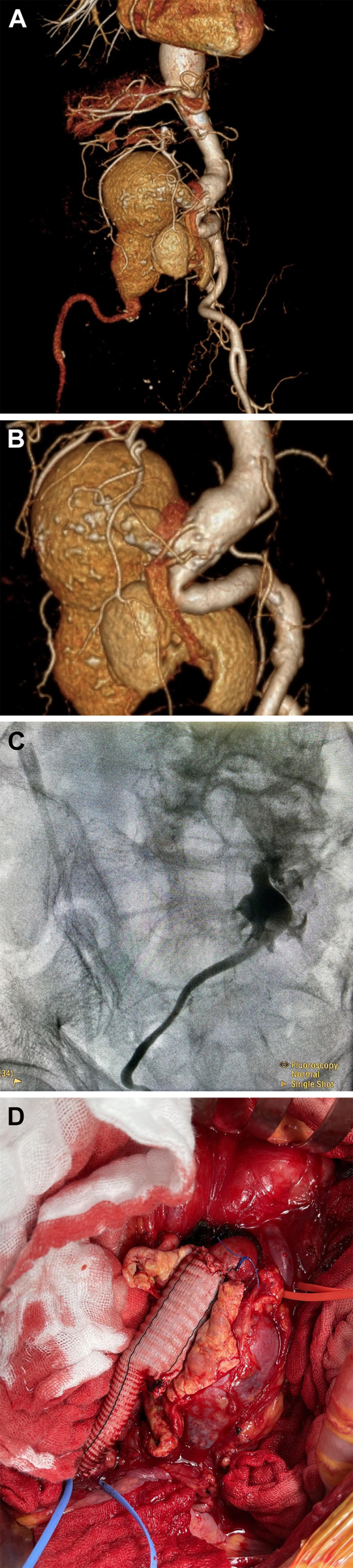

## Figures and Tables

**Cover image:**